# Quantifying individual variability in exposure risk to mosquito bites in the Cascades region, Burkina Faso

**DOI:** 10.1186/s12936-020-03538-5

**Published:** 2021-01-18

**Authors:** Federica Guglielmo, Antoine Sanou, Thomas Churcher, Heather M. Ferguson, Hilary Ranson, Ellie Sherrard-Smith

**Affiliations:** 1 Vector Biology Department, Liverpool School of Tropical Medicine, Pembroke Place, Liverpool, L3 5QA UK; 2 Institute of Biodiversity, Animal Health and Comparative Medicine, University of Glasgow, Glasgow, UK; 3 Centre National de Recherche et de Formation sur le Paludisme, BP 2208, Ouagadougou 01, Burkina Faso; 4 MRC Centre for Global Infectious Disease Analysis, School of Public Health, Imperial College London, St Mary’s Campus, London, W2 1PG UK

## Abstract

**Background:**

The Cascades region, Burkina Faso, has a high malaria burden despite reported high insecticide-treated mosquito net (ITN) use. Human and vector activities outside the hours when indoor interventions offer direct protection from infectious bites potentially increase exposure risk to bites from malaria-transmitting *Anopheles* mosquitoes. This work investigated the degree of variation in human behaviour both between individuals and through time (season) to quantify how it impacts exposure to malaria vectors.

**Methods:**

Patterns in human overnight activity (18:00–06:00) to quantify time spent using an ITN across 7 successive nights in two rural communities, Niakore (N = 24 participants) and Toma (71 participants), were observed in the dry and rainy seasons, between 2017 and 2018. Hourly human landing *Anopheles* mosquito catches were conducted in Niakore specifically, and Cascades region generally, between 2016 and 2017. Data were statistically combined to estimate seasonal variation in time spent outdoors and *Anopheles* bites received per person per night (bpppn).

**Results:**

Substantial variability in exposure to outdoor *Anopheles* bites was detected within and between communities across seasons. In October, when *Anopheles* densities are highest, an individual’s risk of *Anopheles* bites ranged from 2.2 to 52.2 bites per person per night (bpppn) within the same week with variable risk dependent on hours spent indoors. Comparably higher outdoor human activity was observed in April and July but, due to lower *Anopheles* densities estimated, bpppn were 0.2–4.7 and 0.5–32.0, respectively. Males and people aged over 21 years were predicted to receive more bites in both sentinel villages.

**Conclusion:**

This work presents one of the first clear descriptions of the degree of heterogeneity in time spent outdoors between people and across the year. Appreciation of sociodemographic, cultural and entomological activities will help refine approaches to vector control.

## Background

Long-lasting pyrethroid-treated insecticidal nets (ITNs) have proven highly effective in reducing malaria transmission over the past two decades [1] because of the direct protection afforded from the barrier but also, importantly, the insecticidal action to kill mosquitoes, resulting in fewer mosquito bites per person per year across communities [2]. In 2010, the National Malaria Control Programme (NMCP) in Burkina Faso adopted ITNs as the primary preventative strategy against malaria. The country joined many others that follow the World Health Organization (WHO) guidelines to achieve and maintain universal coverage [3] embarking on routine mass distribution every three years. The initiative resulted in household ownership of ITNs rising from 5.6% in 2003 to 89.9% in 2014 and reported use reaching a peak of 67% nationally by 2014 [4] with a corresponding reduction in malaria mortality reported at the national level [5]. Nevertheless, the country remains among those with the highest burden of malaria cases in sub-Saharan Africa, and it has recently experienced a surge in reported cases of infection [5].

Recent entomological evidence has indicated a higher proportion of *Anopheles* human-host feeding attempts are taking place outdoors than previously thought [6, 7]. Debate remains as to whether this is an artefact of the removal of indoor biting mosquitoes through the intense use of indoor interventions or potential changes in *Anopheles* behaviour in several African regions concerning biting time and feeding preferences [8–12]. Consequently, studies on human behaviour have started to investigate patterns of outdoor exposure in malaria-endemic regions to help map human–mosquito interaction [6, 13–15]. The maximum potential efficacy of ITN interventions is ultimately determined by the behaviour of local mosquitoes seeking blood meals and activity of local people moving indoors or to bed, and whether people are using ITNs. A recent systematic review [6] showed that there are sparse published data on human activity (N = 7 studies describing when people are in bed, and 22 studies recording when people move indoors), and fewer paired data noting both mosquito and human behaviours in a matched setting (N = 3 studies) [16–18]. No studies considered changes in individual risk of mosquito bites across nights and through seasons. The lack of understanding about the overlap time in mosquito and human activity restricts the capacity to estimate ITN efficacy yet almost certainly contributes to variability in ITN impact within and between communities [6]. The short-term and respondent-dependent nature of most studies on human behaviour in relation to malaria, which do not quantify nor contextualize human movement, reduce the quality of the data on human activity [8–12, 14]. Identifying groups of people, sites and times of year that render individuals most at risk for “outdoor exposure” can help target outdoor interventions to those most in need.

This work addresses the gap in knowledge on individual- and village-level exposure risk to infectious mosquito bites using an analysis of two rural communities in the Cascades region in south-west Burkina Faso. Data for the time individuals spent outdoors are coupled with entomological data on mosquito biting times taken from the same villages as well as the overall patterns in density and hourly activity observed seasonally across the Cascades region [19]. This approach enables the assessment of local exposure risk to mosquito bites and evaluation of the full potential protection offered by ITNs both within and between individuals across different seasons. Seasonal variations and socio-demographic profiles in relation to individual variation in daily-exposure risk are described. Similar mixed-methods research can be adopted in other settings, in order to relate nocturnal and diurnal activities with cultural, environmental, and socio-economic variations.

## Methods

The Cascades region of Burkina Faso is 18,406 km^2^, with a dry season generally stretching from the end of October to May and a rainy season from June to September. The main human income generation activity is agriculture, particularly cereal crop and legumes, and cotton production. The area has a high burden of malaria infection with prevalence in all ages, reaching 60% [20, 21] to > 80% [22]. The time communities in Niakore and Toma spent indoors overnight, when *Anopheles* mosquitoes that can transmit malaria are most active, is assessed in the analysis. Both human behavioural data and entomological data are then combined to estimate the number of bites received per person per night (bpppn), and the proportion of mosquito bites received outdoors [23, 24] for sociodemographic groups defined by age (under 10-years, 11–20 years, 21–50 years and over 50 years), gender (male and female), and month (April–May; July; October–November). In the analysis, the age groups were identified to provide broadly similar sample sizes in the respective cohorts. These covariates are then used to explain the variation observed in the estimated metrics using regression analyses. Temperature estimates were recorded in situ using two devices: Elitech USB Temperature Data logger, Elitech UK, and Tinytags, Gemini UK.

### Human behaviour

Data on human behaviour were collected within a broader ethnographic approach. Between March 2017 and August 2018, the ethnographer conducted 14 months of participant observation in the region. The ethnographer lived in the communities long-term, and partook in the daily activities of the research participants, including farming, attending social and religious events, and participating in family activities [25, 26]. Participant observation was used to explore the intersection between local lifeways and malaria preventive strategies, and with the additional purpose of limiting response bias, expected in respondent-dependent methods (e.g. surveys, diaries, and other self-reporting approaches) [27]. Additionally, qualitative data on mosquito net use indoors were collected through semi-structured interviews, informal conversations and focus group discussions (Additional file 1: S1). These methods were adopted to investigate how compliance with mosquito net use was understood and performed by the hosting communities in everyday life, focusing on informal mosquito net procurement, intra-household mosquito net allocation, and how participants negotiated treatment-seeking practices for ill family members with their social duties and financial means.

This paper draws on three rounds of structured observations of night-time activities (October–November 2017; April–May, and July 2018). The observations were conducted in two study communities, Niakore and Toma, relying on convenience sampling. Recruited participants resided within 30 metres from the ethnographer’s house and were selected regardless of their gender or age to provide a representation as broad as possible of the nocturnal activities of community members within the confines of haphazard sampling. Adopting a convenience sample ensured consistency in the timing of data collection and research participants’ pool, limiting the structured observations to those participants who lived and stably resided, throughout the weeks of observations, within the established area surrounding the ethnographer’s residence. In Toma, this resulted in 47 individuals (33 females, 14 males) belonging to six different households for observations conducted in October 2017 and April 2018. In July 2018, internal migration of students and farmers increased the pool of participants to 71 (43 females, 28 males), requiring the help of a research assistant. In Niakore, the sample consisted of 24 individuals (14 females, 10 males) from a single household, but this behaviour was found to reflect that of the community more broadly. Age ranges were uneven (e.g. there were only three children aged between 10 and 15 in the sample from Niakore). Across the 7 sampling nights and 3 rounds of data collection, there were a total of 327 and 1070 nightly estimates of the time spent indoors by an individual in Niakore and Toma respectively. Table 1 summarizes sampling night data in each village.

**Table 1:** A summary of the number of sampling nights for human activity recorded in the two sentinel villages; Niakore and Toma

Cohort	Nightly data for Niakore		Nightly data for Toma	
Total	327 (from 24 individuals)		1070 (from 71 individuals)	
Overall	Males (116)	Females (211)	Males (375)	Females (695)
October 2017	–	–	106	230
November 2017	59	112	–	–
April 2018	–	–	78	164
May 2018	57	99	–	–
July 2018	–	–	191	301
Under 10-year olds	28	70	62	137
10–20-year olds	52	58	138	107
21–50-year olds	36	69	137	242
Over 50-year olds	0	14	38	209

### Night-time observations

Structured observations, organized in rounds of one-week, recorded the presence of individuals outdoors throughout the night. The initial observations were conducted after the ethnographer lived in each village for a minimum of 6 weeks to minimize reactivity (the reaction of research participants to the awareness of being observed) [25]. In Toma, observations were conducted in October 2017, April and July 2018; in Niakore, in November 2017 and May 2018. The third round of structured observations in Niakore, scheduled for August 2018, had to be cancelled due to logistical circumstances. Bias in observations was minimized because the ethnographer was embedded within the community, familiar with the participants involved, and able to understand individual nightly patterns of exposure.

Each observation, conducted at intervals of 30 min between 18:00 and 06:00, recorded the time individuals went indoors or exited houses throughout the night. The results do not include shorter instances of time outdoors (e.g. exiting to visit the toilet). A 30-min interval allowed the ethnographer to safely complete the tour of the compounds and confirm the identity of the residents without interfering with any activity they were conducting. At the same time, such an interval ensured that the observations could be standardized, so that people were concluded to be in- or outdoors in a binary fashion, allowing the quantification of human behaviour and the estimation of biting risk. To achieve standardization, this approach scored as ‘outside’ for any given half-hour interval a person who was outdoors at the moment of the observation and engaged in activities classified as ‘labour’ or ‘leisure’. Labour included household chores, farming, the harvesting of caterpillars; leisure referred to resting, sleeping, socializing, participating in social or religious activities. Shorter periods outdoors falling outside this interval, and which may still result in exposure to bites, have been missed and remain a limitation of this approach.

### Mosquito biting behaviour

Mosquito feeding attempts were measured using human landing catches (HLC) [28] across the Cascades region and conducted monthly from 1st October 2016 to 29th December 2019, between the hours of 19:00 to 06:00 [19]. In each village, the collection was carried out twice a month, at two different households each time. Each month, collections were made both inside houses and within the peri-domestic areas. Collectors were between 19 and 30 years of age and randomly assigned to households by pair (2 collectors per household). To avoid bias due to differences in individual attractiveness to mosquitoes, each member of the pair rotated between the indoor or outdoor position every hour. On each collection day, a minimum distance of 30 metres was observed between houses, and 8 metres between the indoor and outdoor collection points of the same household were maintained to avoid biases linked to household location and indoor/outdoor collection points. Mosquitoes were actively collected for 45 min, followed by a 15-min break each hour, as they attempted to feed on the exposed legs of a volunteer.

**Fig. 1:**
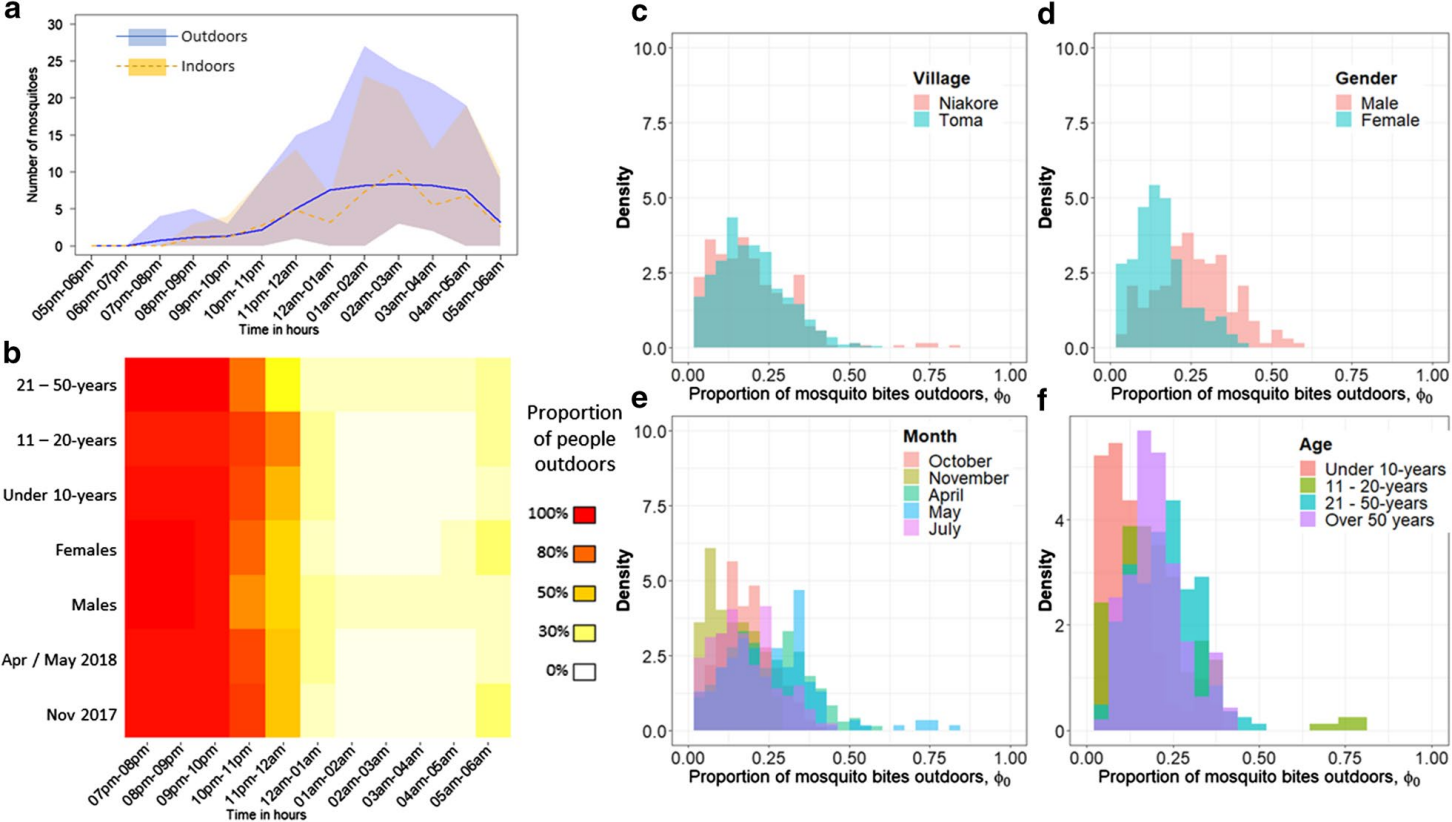
Village-level exploration of mosquito biting risk. **a** The mean (line) and range (polygon of color) hourly number of *Anopheles gambiae s.l.* mosquitoes seeking blood meals indoors (orange) or outdoors (blue) measured using human landing catches taken in Niakore in 2016–2017. **b** The proportion of people who are either outdoors (red) or indoors (white) during the night-time hours as observed using structured observations in Niakore. Cohorts are distinguished by month (sampling nights in Niakore: 10th to 16th November 2017 or April 30th to 6th May 2018), sex (males or females) and age (under 10-years, 11–20-years, or 21–50-years old). The equivalent heat map for Toma is shown in Additional file 1: Fig. S2. Combining these data from both Niakore and Toma, the corresponding range in estimates for the proportion of *An. gambiae s.l.* mosquito bites received outdoors is shown for villages (Niakore, pink; Toma, blue) (**c**), sexes (males, pink; females, blue) (**d**), months (November (gold) and May (blue), Niakore; October (red), April (green) and July (purple), Toma) (**e**), and age cohorts (under 10-years (red), 11 to 20-years (gold), 21 to 50-years (blue), over 50-years (purple)) (**f**)

In Niakore (sampled between 25th October 2016 and 10th November 2017, Additional file 2), the principal malaria vector mosquito complex present was *Anopheles gambiae* *sensu lato* (*s.l*.) (373 mosquitoes indoors and 317 outdoors). Approximately 82% of those molecularly analysed (n = 212 mosquitoes) were *An. gambiae* and 18% were *Anopheles coluzzii* (Additional file 2). Very few other *Anopheles* species were recorded (2 *Anopheles pharoensis* indoors and 4 outdoors; 4 *Anopheles nili* indoors and 1 *Anopheles funestus s.l*. indoors). In Toma, mosquitoes were collected in the dry season (17th, 19th, and 22nd April 2018) and in the wet season (5th, 7th, and 10th September 2018, Additional file 2). The principal species complex was again *An. gambiae s.l.* (566 mosquitoes indoors and 563 outdoors) but molecular distinction of the species complex was not performed. There were 36 *An. pharoensis* (50% indoors), 2 *An. nili* (1 indoors), and a single *Anopheles coustani* and *An. funestus*, both located outdoors. This analysis focused only on *An. gambiae s.l.* (Figure 1a) given that other species were in meagre numbers. Insecticide resistance was tested in Niakore using susceptibility bioassays performed on adult mosquitoes reared from larvae. A total of 58% of 91 *An. gambiae s.l.* tested survived exposure to the discriminatory dose of pyrethroid deltamethrin according to WHO guidelines [29]. In Toma, insecticide susceptibility data were not collected; one limitation is the assumption that the villages sampled in Sanou et al. [19] are representative. The raw mosquito data for Niakore and Toma, included in the broader analysis, are provided in Additional file 2.

There is minimal difference in the number of mosquitoes observed indoors and outdoors in Niakore (373 vs 317, respectively). However, it cannot be established whether this is the result of opportunistic mosquitoes seeking blood meals on exposed volunteers conducting the experiments outside, who would otherwise be indoors (and potentially protected by mosquito nets). The simplifying assumption is that, in the absence of indoor protection, bites received indoors and outdoors are broadly equivalent. This is supported by related work showing relatively equal biting was observed across the Cascades region [19].

The mosquito densities in Cascades region, Burkina Faso, are predictably seasonal and consistent between sample sites, as observed from human landing catch data for *An. gambiae s.l.* (n > 40,000 vector mosquitoes) seeking blood meals outdoors across 12 villages, 324 sampling nights, in 2016 and 2017 (Additional file 1: Fig. S1a; figure adapted from [19]). The pattern of biting activity, i.e. the number of mosquitoes recorded seeking to blood-feed outdoors at each hour of the night, for each week of the year when human activity was observed, was also relatively consistent across months (Additional file 1: Fig. S1b). For each person, for each hour of human activity observations, the time spent outdoors was multiplied by the estimated number of mosquitoes biting at the matched hour and given the predicted seasonal densities corresponding to the week of the year when the human observations were completed. For each night, these hourly estimates of mosquito bites received were summed to estimate the *per night per person* exposure to *Anopheles* bites.

The number of mosquitoes caught during an hourly period (Fig. 1a) is assumed to represent the number of mosquitoes attempting to feed on humans for the same period. In the absence of data, no bites are assumed to occur during the hours for which mosquito bites were not sampled (06:00–19:00). Raw data were converted into the proportion of all mosquito bites received over 24 h, taken indoors (denoted *λ*_*I*_(*t*)) or outside (denoted *λ*_*O*_(*t*)) at hour *(t)* using:

1 $$\lambda_{h} \left( t \right) = \frac{{Sum \, of\, Bites\, at\, hour\left( t \right) for\, location \left( {inside\, or\, outside} \right)}}{Sum\, of\, bites\, for\, all\, hours\, for\, both\, locations}$$

where subscript *h* indicates whether bites are taken indoors (*h *=1) or outdoors (*h *=0) [30].

For different sociodemographic groups (age, gender), months and villages, the proportion of *An. gambiae s.l.* bites received outdoors was assessed to understand the potential of indoor intervention and the protection gap remaining for outdoor control. The observational data on people movement are summarized by gender, age and month to estimate the varying proportion of *An. gambiae s.l.* bites received outdoors (*ϕ*_*O*_) following [23, 30]:

2 $$\varPhi_{O} = 1 - \frac{{\mathop \sum \nolimits_{t} p_{I} \left( t \right)\lambda_{I} \left( t \right)}}{{\mathop \sum \nolimits_{t} \left( {\left( {1 - p_{I} \left( t \right)} \right)\lambda_{0} \left( t \right) + p_{I} \left( t \right)\lambda_{I} \left( t \right)} \right)}}$$

where *p*_*I*_(*t*) is the proportion of people inside at hour (*t*), *λ*_*I*_(*t*) is the biting rate indoors at hour (*t*), and *λ*_*O*_(*t*) is the biting rate outdoors at hour (*t*). The analysis of variance results using the Cascades data to estimate the proportion of mosquito bites received indoors and outdoors is provided (Additional file 1: Table S1) and histograms of the Niakore specific data are presented in Fig. 1 (Table 2).

**Table 2:** The proportion of mosquito bites received indoors (1 – *Φ*_*O*_, Eq. 2) as estimated by the overlapping activity of humans and mosquitoes in Niakore and Toma

Cohort	Niakore			Toma		
	Mean	Median	Range	Mean	Median	Range
Males	0.74	0.79	(0.51–0.86)	0.68	0.69	(0.48–0.81)
Females	0.78	0.83	(0.64–0.87)	0.83	0.83	(0.74–0.90)
Under 10 years	0.811	0.86	(0.64–0.91)	0.89	0.87	(0.79–0.97)
11 to 20 years	0.73	0.81	(0.24–0.86)	0.78	0.78	(0.63–0.89)
21 to 50 years	0.76	0.77	(0.68–0.86)	0.72	0.73	(0.54–0.86)
Over 50 years				0.77	0.79	(0.63–0.87)
Oct/Nov 2017	0.83	0.83	(0.77–0.91)	0.79	0.80	(0.63–0.89)
Apr/May 2018	0.70	0.71	(0.24–0.86)	0.72	0.72	(0.48–0.93)
July 2018				0.81	0.80	(0.62–0.97)
Overall summary						
Males	0.71	0.70	(0.48–0.86)			
Females	0.81	0.83	(0.64–0.90)			
Under 10 years	0.86	0.87	(0.64–0.97)			
11 to 20 years	0.76	0.78	(0.24–0.89)			
21 to 50 years	0.74	0.75	(0.54–0.86)			

These estimates are determined from village-specific data on human activity moving indoors or outdoors throughout a 12-h period overnight and mean hourly mosquito blood-feeding behavior human landing catch HLC data collected across the Cascades region in 2016–2017. The mean, median and range in the proportion of mosquito bites received indoors for different cohorts of the community are noted. Corresponding estimates using the Niakore specific HLC data are provided in Additional file 1: Table S1

### Statistical analysis

At the individual level, the association between age, sex, and month was investigated in relation to: (i) the number of hours spent indoors per person per night (*y*_*A*_), or (ii) the number of bites received per person per night (*y*_*B*_) using two generalized linear mixed-effects models (GLMM) that took the structure:

3 $$y_{i} \sim D\left( {f\left( {\eta_{i} } \right),\theta } \right)$$

4 $$\eta = {\mathbf{X}}\beta + {\mathbf{Z}}u$$

where the linear predictor *η* (transformed by the inverse link function *f* and assuming a Gaussian distribution *D*) was fitted to the data with age, month (distinct between villages) and gender included as explanatory variables in matrix **X**, and repeated measures for each individual *i* included as random effects in **Z**. The parameter $\theta$ is the standard deviation. Parameters *β* and *u* are coefficients at the population level and group level (for random effects) respectively. Given the observations made on distinct individual behaviours between months, interactions between age and month (which was later dropped as insignificant), and sex and month were included in the model exploring the number of bites received per person per night. Both models were fitted in a Stan computational framework (http://mc-stan.org/) accessed with the ‘brms’ package [31]. All data are provided in Additional file 2.

Finally, differences in the predicted number of bites received per person across weeknights were tested independently for each month to understand how social activity might be driving differences in exposure risk. A general linear model (‘stats’ package, R [32]) was fitted to the log-transformed number of bites received per person per night (*y*_*C*_) with weeknight (Monday to Sunday) and gender included as explanatory variables (Additional file 1: Table S2, Fig. S3).

## Results

### Human activity

There were sociodemographic and seasonal differences in the time spent indoors or outdoors (Fig. 1b). Both gender and month were strongly associated with differences in the proportion of time spent indoors per person per night. The statistical model (GLMM) estimated that males spent a median of 65 min (95% CrI = 37 to 90 min) more time outside per night than females. The pattern was observed in both Niakore (Fig. 1b) and Toma (Additional file 1: Fig. S2).

There were village-specific patterns of behaviour. In Niakore, all observed individuals remained outdoors until 22:00 (Fig. 1b); in Toma, individuals started to go indoors after 21:00 (Additional file 1: Fig. S2). The GLMM indicated that people are inside for a median of 29 min (95%CrI = − 2 min to 61 min) longer in Toma than Niakore: an earlier retreat indoors was accompanied by an earlier exit outdoors in the morning (contrast Fig. 1b with Additional file 1: Fig. S2). In Toma, some participants spent the whole night outdoors during April; one male remained outdoors all night on all 5 observation nights, 4 other males and 1 female remained outside throughout the night on 3 of the 5 observation nights. Under 10-year olds tended to spend more time inside than other age cohorts (median 32 min, 95% CrI = 6 to 58 min), and there was a gradual trend toward more time outdoors as age increased, which corresponded to a higher proportion of bites received outdoors for older age groups (Fig. 1f).

People in Toma spent 69 min longer outside in April (95% CrI = 57 to 80 min) relative to July and October (Additional file 1: Fig. S2). Similarly, people in Niakore spent more time outdoors in May than in November (Fig. 1b). These observations were associated with cultural activities detailed below, e.g. foraging caterpillars, farming, and attending ceremonies during the funerary season. The Bayesian model diagnostics and predictions are provided in Additional file 2.

Round 1 (Oct/Nov 2017): outdoor/indoor patterns were observed toward the end of the agricultural season, dedicated to the harvest. Seasonal workers resumed their temporary or urban employment, either leaving the village or adapting to local factories’ three-shift work rotas (6:00 to 14:00; 14:00 to 22:00; 22:00 to 6:00). During these dry months, the lower temperatures (between 21 °C and 23 °C in the evening) meant that adults were less likely to spend the whole night outside. On average, 422 min (range: 0–720 min) were spent indoors by community members from Toma in October and 440 min (range: 330 min–750 min spent indoors) by community members from Niakore in November.

Round 2 (Apr/May 2018): activities were observed during the dry season and at the end of the funerary season. Families celebrated the life of those who passed away during the previous months through 3-day long celebrations, held outdoors and lasting well into the night [33]. Children were still attending school, and factory work was intermittent. Heat and ritual celebrations motivated higher levels of outdoor activities in both communities. Individuals of both sexes, but particularly males, spent time outdoors in leisure (socializing or resting) until temperatures indoors lowered enough to allow sleep. On average, 385 min (range: 0–570 min) were spent indoors by community members from Toma in April, and 361 min (range: 0–720 min) by community members from Niakore in May.

Round 3 (July 2018): observations took place during the rainy season, when rural areas register the highest number of permanent residents. In Toma, where farming land was scarce due to government expropriation in the 1970s, those with access to land (either their own or as paid laborers, working the fields belonging to others) rose earlier and engaged in a range of agricultural activities until late in the day. On one night, between 02:00 and 04:30, which coincides with peak mosquito blood-seeking activity (Additional file 1: Fig. S1b), as many as 12 women and 2 children (one male and one female) were outdoors foraging for caterpillars (*Cirina butyrospermi*). An important source of fat and protein, caterpillars were eaten freshly cooked, dried out and preserved for months, or sold in markets. Feeding exclusively on shea trees (*Vitellaria paradoxa*) [34], caterpillars were abundant in Toma but absent in Niakore. On average, 465 min (range: 0–630 min) were spent indoors by community members from Toma in July.

Temporary residents, students and relatives who attend school or live elsewhere, returned to their respective villages between June and September to spend the holidays or farm. In Toma, this phenomenon doubled the pool of participants aged 11–25-years in the July survey, when students remained outdoors later (22:30–04:30), partaking in leisure activities.

### Mosquito activity

Sanou et al. [19] comprehensively analysed the mosquito data in the Cascades region, and the seasonal densities predicted by that study for *An. gambiae s.l.* host seeking outdoors are shown in Additional file 1: Fig. S1a. Mosquito densities in Niakore varied across months. The highest densities were sampled in September 2018, when an average 2.82 and 3.51 *An. gambiae s.l.* per hour were caught in HLCs indoors and outdoors, respectively (maximum catches per hour indoors and outdoors were 7.7 and 9 recorded in September 2018 and June 2018, respectively) (Additional file 1: Fig. S1a). October 2016 had the lowest average number of *An. gambiae s.l.* seeking a blood meal indoors (0.21 per hour) and outdoors (0.26 per hour), although these numbers were higher in October 2017 (0.62 and 0.92 *An. gambiae s.l*. mosquitoes seeking a blood meal per hour indoors and outdoors, respectively). Mosquito activity was highly variable between nights (N = 2 sampling nights per month) and households (N = 2 houses per month) [19]. The patterns from Niakore broadly reflected the timing of biting trends seen across the Cascades region (Additional file 1: Fig. S1b).

### Proportion of mosquito bites received outdoors

Differences in human behaviour resulted in substantial variability in the estimated proportion of *An. gambiae s.l.* bites that people received outside. Overall there were no differences between villages (Analysis of Variance, AOV F-stat = 0.56, *p *= 0.454, Fig. 1c). However, the proportion of mosquito bites received outdoors varied by sex (AOV F-stat = 24.0, adj-R^2^ = 0.261, *p* < 0.001, Fig. 1d), and particularly by month (AOV F-stat = 34.6, adj-R^2^ = 0.16, *p* < 0.001, Fig. 1e) and with a person’s age (AOV F-stat = 10.2, adj-R^2^ = 0.19, *p* < 0.001, Fig. 1f) (Table 2 and Additional file 1: Table S1). The estimated proportion of *An.* *gambiae* *s.l.* mosquito bites received outdoors in Niakore (median of 21.3% and 17.2%, range 3.5–76%, and 6.3–74.4%, using Cascades HLC data and Niakore village-level HLC data, respectively) was higher than median estimates from systematic reviews that have been predicted previously for African communities, although within the estimated range (median bites outdoors: 11% [23, 30], range 0–77%, [6]).

### Number of bites received per person per night

**Fig. 2:**
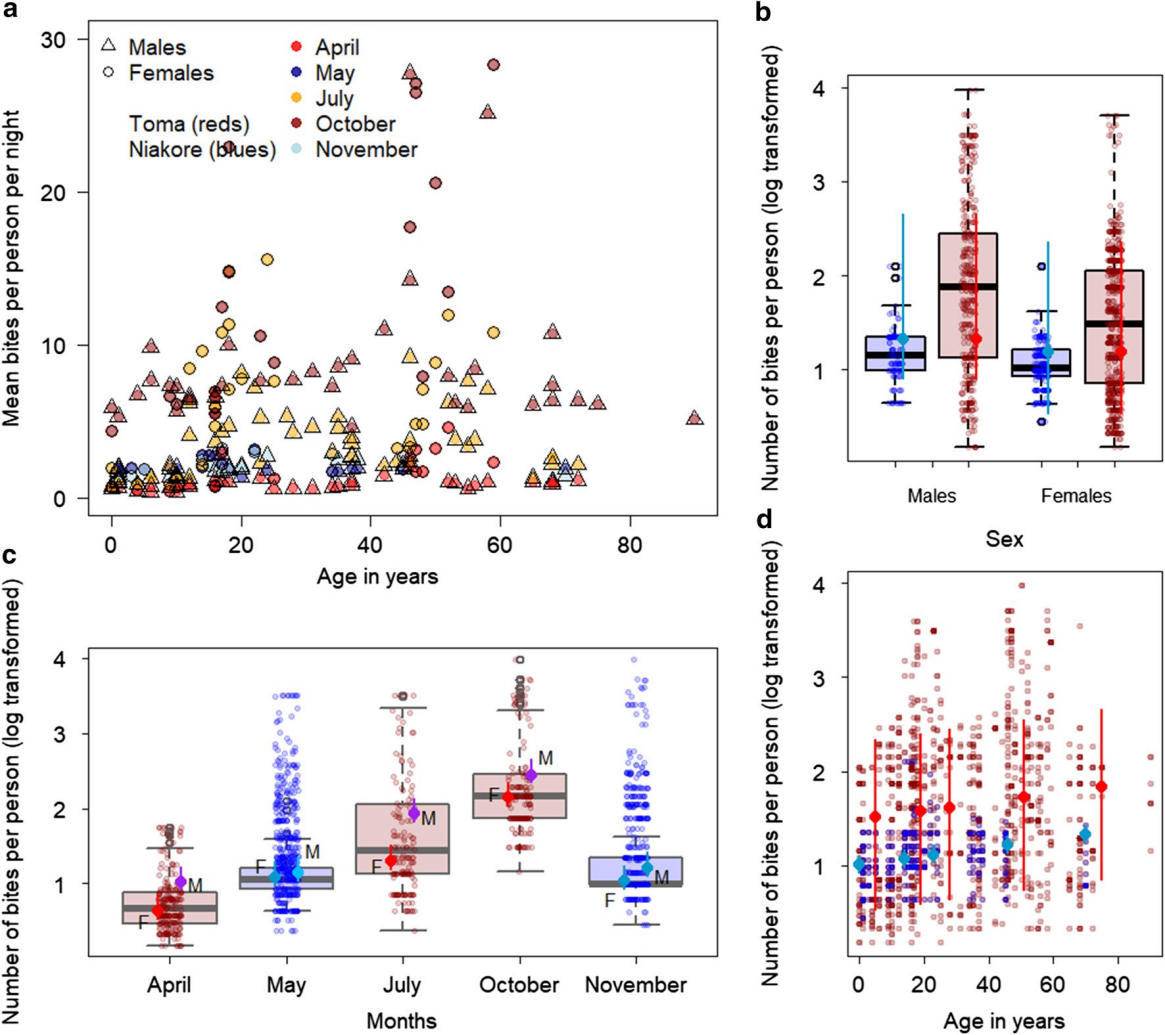
Variation in estimated bites from *Anopheles gambiae s.l.* mosquitoes per person per night in two villages in Burkina Faso. **a** The summary data (mean bites per person per night) explored using the generalized linear model with gender, month and age cohort as explanatory variables and individual as a random effect (data are provided in Additional file 2). Males (open triangles) are predicted to receive the highest number of bites in October (dark red) and July (orange), both representing individuals from Toma. Fewer bites per person per night are predicted for Niakore (blues) although, more bites are received in May (dark blue) than November (light blue). **b**–**d** The model predictions (points show median model estimate, with line segments, 95% Credible Intervals) overlaid onto the data (boxplots show median, central line; 50-percentiles, edge of box; and 95-percentiles, edge of segments, with jittered points marking individual data). Blue dots represent the observed data for Niakore and red dots those for Toma. **b** The interaction between sex and month is shown in the model predictions (offset points and segments for females, left of center, and males, right of center). Data are log_e_ transformed

The GLMM explained 71.83% of the variation in the predicted number of bites per person per night using gender, month and age as covariates and individual as a random effect (Additional file 2, F-stat = 72.1, adj-R^2^ = 0.718, *p *< 0.001, Fig. 2). The greatest number of bites were predicted in October, mean 9.39 (95% Credible intervals: 7.77–11.38) bites per person per night, and fewest bites were predicted in April, mean 1.41 (95% CrI 1.03–1.88) bites per person per night, even though outdoor activity was most significant in April (Fig. 2a, c) stressing the importance of *Anopheles* density for exposure risk.

Males (4.73, 95% CrI 3.69–6.05 bites per person per night) were predicted to receive more bites than females (3.13, 95% CrI 2.49–3.91 bites per person per night), which reflects the greater time spent outdoors by males at times in the year when *Anopheles* densities are higher (Fig. 2c), in both Toma and Niakore. There was also a general increase in risk with age cohort; infants, median age 0 years, were predicted to have 3.25 (95% CrI 2.52–4.20) bites per night compared to 3.53 (95% CrI 2.85–4.41), 3.73 (95% CrI 3.05–4.60), 4.26 (3.42–5.28) and 4.88 (3.64–6.40) bites per night for median ages of 14-, 23-, 46-, and 70-years respectively (Fig. 2d). For Bayesian model diagnostics and predictions, see Additional file 2.

**Fig. 3:**
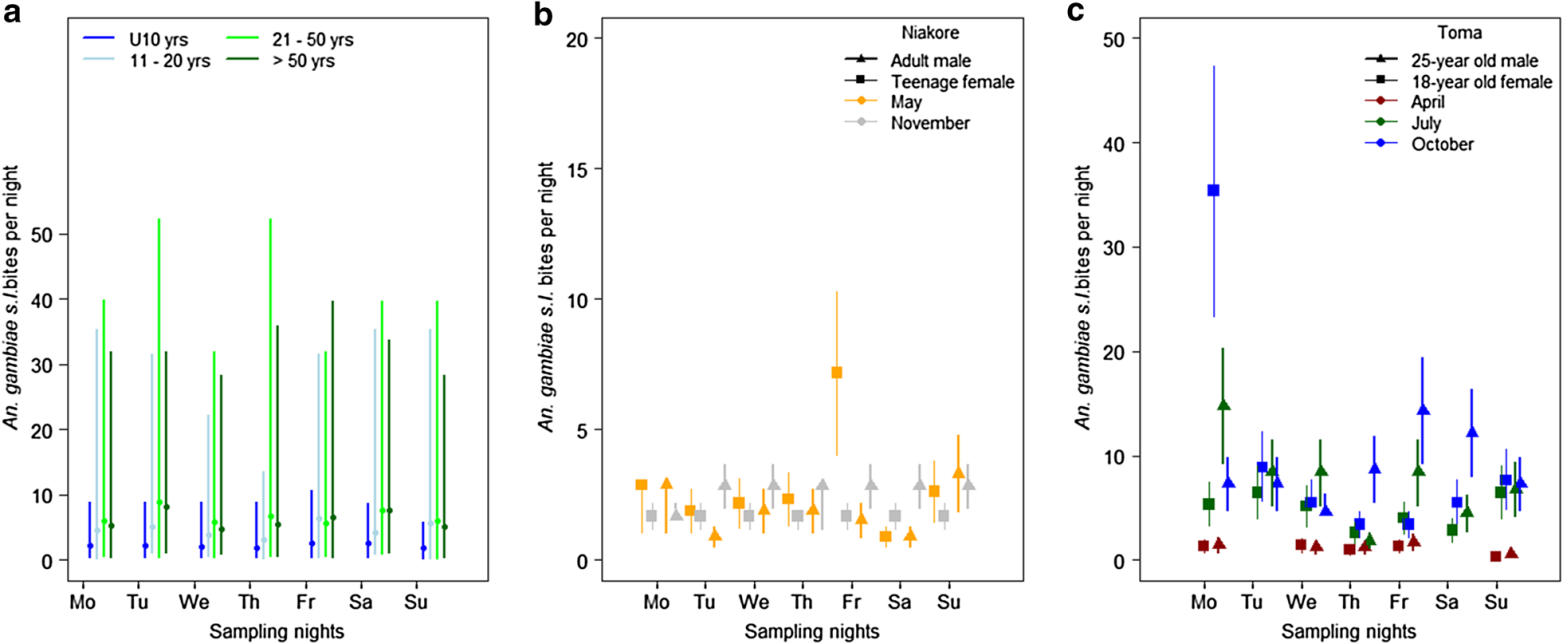
Variation in exposure risk of individuals across sampling nights. The estimated average number of *Anopheles gambiae* s.l. bites per person per night using the time spent outdoors by humans observed in 2017–2018 and the number of mosquitoes attempting to feed outdoors from Cascades region human landing catch data recorded in 2016–2017 [19]. **a** The number of bites across weeknights for the respective age cohorts: under 10-year olds (blue), 11–20-year olds (light blue), 21–50-year olds (light green) and over 50-year olds (dark green). Under 10-year olds consistently receive few bites relative to the older population. The estimated 95% confidence interval (using 95% CI from HLC data, Sanou et al. pers. comm.) and mean number of *An. gambiae s.l.* bites (points) received for two people in Niakore (**b**) and Toma (**c**) respectively across different sampling months and weeknights. Additional file 1: Fig. S3 provides individual data for the Niakore and Toma populations observed in this study

There was considerable variation across nights in each month (Fig. 3), driven specifically by differences in human behaviour. The same individual is not always predicted to receive the most bites, although some individuals are consistently exposed to higher numbers of bites (Additional file 1: Fig. S3). The mean and 95% confidence intervals in the predicted number of *An. gambiae s.l.* bites received for a male and female from Niakore (Fig. 3b) and Toma (Fig. 3c) demonstrate the heterogeneity of exposure across nights. For example, one female in Toma spent only 3 h indoors on one night, which corresponded to a predicted 35.4 (95% confidence intervals 23.3 – 47.3) *An. gambiae s.l.* bites through the night. The greatest variation in the number of bites received per person per night was observed for a male in the 50 and over cohort in October, who was estimated to be at risk to 2.19 bites on one night and ~ 52.24 bites on the other 2 nights. In Toma, both males and females were less at risk on Sunday nights in April (p < 0.0001, see Additional file 1: Table S2), and Wednesday nights in October (p = 0.023, Additional file 1: Table S2), whilst there were no significant differences in risk across weeknights for July (Additional file 1: Table S2). In Niakore, Monday was the riskiest night in May (p < 0.05, Additional file 1: Table S2) and people were least at risk on Saturday (Fig. S3), which contrasted with November when significantly more bites were predicted for Friday, Saturday and Sunday across the community (p < 0.05, Additional file 1: Table S2), reflecting differences in communities’ social lives. Data are summarized in Additional file 1: Fig. S3.

## Discussion

Socio-demographic, economic, and environmental circumstances [13, 14] (Guglielmo et al. in press) influence the activities conducted outside the hours when ITNs can provide protection. As the analysis demonstrates, nightly differences in *An. gambiae s.l*. biting rates can be substantial across any given week because of differences in human outdoor activity. Whilst seasonal patterns are driving mosquito densities and contribute toward predicting exposure at the community level, outdoor activities are equally crucial to determine biting risk at an individual level. To give some indication of the relative magnitude of these effects, within a year, mosquito densities can drive predicted bites from 0 (in the dry season) to over 80 bites per person per night in the peak *Anopheles* season (Fig. S1a). In comparison, hours spent outside during the transmission season (July and October) suggest that human behaviour can alter risk to a similar extent, from 2.2 to over 50 bites per person per night. These data show that nightly exposure to biting for any individual changes daily (Fig. 3, Additional file 2: Fig. S3) with potentially tenfold differences in exposure risk common across a single sampling week.

Previous studies have quantified average community exposure to mosquito biting through the overlap time in hourly activity of mosquitoes and humans [6, 23, 24, 35]. These studies provide a general understanding of exposure risk to mosquito bites summarized as the proportion of bites received indoors (Table 2), in bed, or outdoors, but until recently did not capture the evident individual heterogeneity contributing to this risk [15]. It is reasonable to posit that receiving more *Anopheles* bites per night would correspond to an increased risk of malaria transmission. Heterogeneous biting has been shown previously to alter the reproductive number for malaria, R_0_, by an order of magnitude [36]. Some individuals are predicted to be bitten extensively when consistently spending time at night outdoors. Nevertheless, individual behaviours tended to differ nightly, proportionally altering risk (Fig. 3), which makes a case for malaria control efforts to invest in personal protection whilst people engage in outdoor activities. This framework may be particularly useful if social activities render some weeknights riskier (Additional file 1: Table S3). Spatial variation within communities is not explicitly considered, though differences between malaria transmission risk have been previously observed across households given, for example, proximity to breeding sites [37].

The distribution of sporozoite-positive bites has been shown to target a small proportion of the population with a few individuals receiving multiple infectious bites [38]. This study does not consider sporozoite rates of mosquitoes feeding at different hours of the night nor heterogeneity in community immunity patterns. As such, this work cannot determine how these different biting rates might contribute to malaria transmission. Nonetheless, the seasonal densities in sporozoite rates for the Cascades region suggest that bites received in October and November are most likely to be infectious (Sanou et al., pers. commun.). In Burkina Faso, sporozoite rates are very high [38]; in the Cascades region, the *P. falciparum* sporozoite rate in *An. gambiae* sensu stricto ranged from 3.64 to 7.66% [19]; in Niakore, more specifically, sporozoite rates ranged from 3 to 12% (Sanou et al. pers. comm.). These data suggest that increased exposure to *Anopheline* mosquito bites is highly likely to correlate with an increased infection risk. Given the high malaria prevalence in the area (approximately 60%, [20, 21] to > 80% [22]), a correlation between people’s different behaviours and infection levels (see the uncertainty in the relationship between malaria prevalence and incidence as prevalence becomes very high [39]) would not necessarily be expected, although it would be interesting to explore case data, were they available, to understand repeated incidence in higher-risk individuals. Regardless, these observations contribute to the debate on why vector control in Toma and Niakore, and more broadly across the Cascades region, is falling short and may continue to do so even were higher ITN usage achieved.

High levels of outdoor human activity in April (Niakore) and July (Toma), compared to other survey months, were attributed to indoor spaces being too hot for sleep [40, 41] and to undergoing leisure or work activities, which drove higher biting rates in July (Fig. 2c). Both rainfall and agricultural activity also drove night-time biting risk in Niakore and Toma. As expected, mosquito densities in Niakore peaked in the rainy months (increasing from June through to late September), with mosquito host-seeking behaviour mirroring that observed previously for *An.* *gambiae* *s.l.* in West Africa [23, 42–44], with few early evening bites and peak biting from around 23:00 until the early morning (Fig. 1a). However, biting risk was enhanced in July because people spent more time outdoors compared to other survey months. In May, extensive outdoor activity by the community was less problematic given low mosquito densities in this season [19]. Some previous work has documented that children contribute more parasites to mosquitoes [22], although not always [45]. Adults have been observed previously to receive a more significant number of bites which balances this contribution to the infectious reservoir [22]. This work clearly shows that the greater outdoor activity of people 20-years and over is also associated with receiving more bites (Figs. 1f, 2d, 3a) and may be an additional reason why adults are bitten more often. Human and mosquito activity data have been combined previously to understand exposure risk to vector-borne diseases in subgroups of populations [13, 14, 17, 23, 41].

The main limitations of the methods adopted here are the inability to sample the population randomly and to increase the sampling size of data collated due to cost and time commitment. For these reasons, surveys are often preferred [14, 46, 47, 49], although this choice bears its own shortcomings [50]. Direct observations reduce the bias associated with changes in community behaviours that could misrepresent community activity; however, the effects of short-term observations [13, 17] and their logistics [41, 50, 51] on reactivity in similar studies remain mostly unaddressed. In this study, the ethnographer observed key behaviours that alter who may be at risk of mosquito bites, and which would not have been captured by surveys. For example, in July, women and children collected caterpillars during the early hours of the morning—a key resource for the community in Toma. Survey results are also, and in different ways, very dependent on the month in which they are conducted: in June through September, there is an increased risk to young adults returning to families driven by both adolescent age cohorts staying up later and changes in ITN availability for these people, coupled with increased mosquito densities [19]. Work-related commutes [52] and internal migration [53] are known to affect ITN availability. One of the reasons that surfaced from long-term participant observation is because census data might be collected when people are away: ITN distributions can fall short because additional ITNs for transient community members are not provided.

Across the African continent, the Anopheline mosquitoes that are responsible for malaria transmission are increasingly able to survive exposure to previously lethal doses of pyrethroid insecticide. Pyrethroids represent the active ingredient traditionally used in ITNs, and pyrethroid resistance is cited as a potential reason for the resurgence in malaria cases observed in many countries across the continent [54, 55]. Although pyrethroid resistance is not addressed in the current analysis, it has been previously shown that the presence of resistant mosquitoes interacts with outdoor mosquito bites to exacerbate the challenge for control [6]. If mosquitoes are not dying, the time people spend outside becomes more vital to determining the efficacy of indoor based interventions [6]. In areas like Cascades region, Burkina Faso, it will be critical to reducing mosquito densities to recover control. Spatial repellents [56], endectocides [57], attractive targeted sugar baits [58], larval source management (LSM) strategies [59, 60], and potentially gene drive technologies [61] could all be beneficial in these areas; with perhaps the exception of LSM, these are yet to be thoroughly tested. In the immediate term, topical repellents may benefit people whilst out overnight [48] and community engagement to minimize breeding habitat around human settlements [62] may generally lower mosquito densities and thus minimize exposure risk.

There is evidence that there can be significant interannual differences in vector dynamics [63, 64]. Two key limitations of this study are the assumptions that entomological data from across the Cascades region is representative of Niakore and Toma specifically, and that one year reflects the next. The broad agreement between the Niakore and Cascade region entomology is reassuring (Additional file 1: Fig. S1). Parallel collection of entomological and human activity data would need to be carefully considered to avoid the former biasing activity of community members. There will be nightly variation in mosquito activity being overlooked given our sampling strategy (2 nights and 2 households per village per month). Sampling across multiple consecutive nights could provide a better understanding and probably intensify heterogeneity in biting and associated risks of malaria transmission. Based on long-term observations by the ethnographer, another assumption is that people move indoors to go directly to bed and are protected by an ITN. The decision to limit observations to the outdoors aimed at minimizing reactivity and observer’s bias and at respecting the privacy of the research participants. As such, this analysis does not account for any indoor biting, which would increase the estimated *An. gambiae s.l.* bites per person per night. The quality, type, or age of ITNs used in the sentinel villages is not known and this will also impact ITN efficacy and personal protection [65]. In this study, representativeness was substituted by richness of data, focusing on 2 villages to generate conclusions about individual risk.

## Conclusion

Outdoor exposure risk to mosquito bites is occurring in Niakore and Toma and varies substantially between individuals and across weeknights dependent on outdoor activity. How much this contributes to the overall malaria burden and failure to control the infection in this area of the Cascades is yet to be confirmed. These results support the hypothesis that outdoor exposure to *Anopheles* biting, along with other factors including pyrethroid resistance, is playing a role in the observed low impact of indoor vector interventions in the Cascades region. Protection from vector bites depends on both night and day activities that are influenced by environmental, cultural, and socio-economic circumstances. While the data collected in this study are not strictly generalizable, they are transferable and contribute to understanding ITN potential. This approach is particularly relevant in areas where behavioural changes are observed among local vector populations, and where human migration and outdoor sleeping are recurring phenomena. Social gatherings, work patterns, age, social status, and internal migration due to schooling or farming all contribute to mosquito bite exposure in different ways throughout the year. To be sustainable, interventions must account for the heterogeneity in host activity as well as their vectors, acknowledging the context-based limitations intrinsic to indoor-based applications.

## Key messages

- Exposure risk to *Anopheles* bites varied by tenfold on any given night when individuals spend more time outdoors.
- Seasonal patterns in *Anopheles* densities drive biting risk but this risk is substantially reduced as people spend longer indoors in a protected environment.
- Males are predicted to be at higher risk of exposure to outdoor *Anopheles* bites than females due to longer outdoor activity in the evening or through the night.
- Adults over 21-years are at higher exposure risk to outdoor *Anopheles* bites than younger people in the same community due to longer time spent outdoors nightly.
- Consideration of these heterogeneities in time spent outdoors could help identify the most vulnerable groups for targeted outdoor vector control interventions.

## Supplementary information

**Additional file 1.** S1.1 Interview questions asked of participants. S1.2 Guidelines of topics addressed in informal focus group discussions. S1.3 Template followed for structured observations. S1.4 Summary of methods used to estimate biting risk per person per night.

**Additional file 2.** Raw data for GLM 1: Predicting the time spent indoors per person per night. Raw data for GLM 2: Predicting the bites received per person per night. Raw mosquito data: indoors. Raw mosquito data: outdoors. Output for GLMM 1, model statistics and predictions. Output for GLMM 2, model statistics and predictions.

## Data Availability

The datasets used and/or analysed during the current study are provided in Additional file 2. Authors can provide raw observational data on human activity upon request.

## Abbreviations

AOV: Analysis of variance
Bpppn: Bites per person per night
ITN: Long-lasting pyrethroid treated mosquito nets
HLC: Human landing catch
GLMM: Generalized linear mixed-effects models

## References

[1] Bhatt S, Weiss DJ, Cameron E, Bisanzio D, Mappin B, Dalrymple U (2015). The effect of malaria control on *Plasmodium falciparum* in Africa between 2000 and 2015. Nature.

[2] Thomas MB, Read AF (2016). The threat (or not) of insecticide resistance for malaria control. Proc Natl Acad Sci USA.

[3] WHO (2017). Achieving and maintaining universal coverage with long-lasting insecticidal nets for malaria control.

[4] Samadoulougou S, Pearcy M, Yé Y, Kirakoya-Samadoulougou F (2017). Progress in coverage of bed net ownership and use in Burkina Faso 2003–2014: evidence from population-based surveys. Malar J..

[5] WHO (2018). World malaria report 2018.

[6] Sherrard-Smith E, Skarp JE, Beale AD, Fornadel C, Norris LC, Moore SJ (2019). Mosquito feeding behavior and how it influences residual malaria transmission across Africa. Proc Natl Acad Sci USA.

[7] Killeen GF, Kiware SS, Okumu FO, Sinka ME, Moyes CL, Massey NC (2017). Going beyond personal protection against mosquito bites to eliminate malaria transmission: population suppression of malaria vectors that exploit both human and animal blood. BMJ Glob Health..

[8] Reddy MR, Overgaard HJ, Abaga S, Reddy VP, Caccone A, Kiszewski AE (2011). Outdoor host seeking behaviour of *Anopheles gambiae* mosquitoes following initiation of malaria vector control on Bioko Island, Equatorial Guinea. Malar J..

[9] Russell TL, Govella NJ, Azizi S, Drakeley CJ, Kachur SP, Killeen GF (2011). Increased proportions of outdoor feeding among residual malaria vector populations following increased use of insecticide-treated nets in rural Tanzania. Malar J..

[10] Padonou GG, Gbedjissi G, Yadouleton A, Azondekon R, Razack O, Oussou O (2012). Decreased proportions of indoor feeding and endophily in *Anopheles gambiae s.l.* populations following the indoor residual spraying and insecticide-treated net interventions in Benin (West Africa). Parasit Vectors..

[11] Moiroux N, Gomez MB, Pennetier C, Elanga E, Djènontin A, Chandre F (2012). Changes in *Anopheles funestus* Biting Behavior Following Universal Coverage of Long-Lasting Insecticidal Nets in Benin. J Infect Dis.

[12] Thomsen EK, Koimbu G, Pulford J, Jamea-Maiasa S, Ura Y, Keven JB (2016). Mosquito behaviour change after distribution of bednets results in decreased protection against malaria exposure. J Infect Dis.

[13] Finda MF, Moshi IR, Monroe A, Limwagu AJ, Nyoni AP, Swai JK (2019). Linking human behaviours and malaria vector biting risk in south-eastern Tanzania. PLoS ONE.

[14] Monroe A, Moore S, Koenker H, Lynch M, Ricotta E (2019). Measuring and characterizing night time human behaviour as it relates to residual malaria transmission in sub-Saharan Africa: a review of the published literature. Malar J..

[15] Fornace KM, Alexander N, Abidin TR, Brock PM, Chua TH, Vythilingam I (2019). Local human movement patterns and land use impact exposure to zoonotic malaria in Malaysian borneo. Elife..

[16] Seyoum A, Sikaala CH, Chanda J, Chinula D, Ntamatungiro AJ, Hawela M (2012). Human exposure to anopheline mosquitoes occurs primarily indoors, even for users of insecticide-treated nets in Luangwa Valley, South-east Zambia. Parasit Vectors..

[17] Geissbühler Y, Chaki P, Emidi B, Govella NJ, Shirima R, Mayagaya V (2007). Interdependence of domestic malaria prevention measures and mosquito-human interactions in urban Dar es Salaam, Tanzania. Malar J..

[18] Cooke MK, Kahindi SC, Oriango RM, Owaga C, Ayoma E, Mabuka D (2015). ‘A bite before bed’: exposure to malaria vectors outside the times of net use in the highlands of western Kenya. Malar J..

[19] Sanou A, Guelbego WM, Nelli L, Toé HK, Zongo S, Ouerdraogo P (2019). Evaluation of Mosquito Electrocuting Traps as a safe alternative to the Human Landing Catch for measuring human exposure to malaria vectors in Burkina Faso. Malar J..

[20] Tiono AB, Ouédraogo A, Ouattara D, Bougouma EC, Coulibaly S, Diarra A (2018). Efficacy of Olyset Duo, a bednet containing pyriproxyfen and permethrin, versus a permethrin-only net against clinical malaria in an area with highly pyrethroid-resistant vectors in rural Burkina Faso: a cluster-randomised controlled trial. Lancet.

[21] Pombi M, Calzetta M, Guelbeogo WM, Manica M, Perugini E, Pichler V (2018). Unexpectedly high *Plasmodium* sporozoite rate associated with low human blood index in *Anopheles coluzzii* from a LLIN-protected village in Burkina Faso. Sci Rep..

[22] Gonçalves BP, Kapulu MC, Sawa P, Guelbéogo WM, Tiono AB, Grignard L (2017). Examining the human infectious reservoir for *Plasmodium falciparum* malaria in areas of differing transmission intensity. Nat Commun..

[23] Killeen GF, Kihonda J, Lyimo E, Oketch FR, Kotas ME, Mathenge E (2006). Quantifying behavioural interactions between humans and mosquitoes: evaluating the protective efficacy of insecticidal nets against malaria transmission in rural Tanzania. BMC Infect Dis.

[24] Huho B, Briët O, Seyoum A, Sikaala C, Bayoh N, Gimnig J (2013). Consistently high estimates for the proportion of human exposure to malaria vector populations occurring indoors in rural Africa. Int J Epidemiol.

[25] Bernard RH (2011). Research Methods in Anthopology. Qualitative and Quantitative approaches.

[26] Pigg SL (2013). On sitting and doing: ethnography as action in global health. Soc Sci Med.

[27] van de Mortel T (2008). Faking it: social desirability response bias in self-report research. Aust J Adv Nurs..

[28] Bradley J, Lines J, Fuseini G, Schwabe C, Monti F, Slotman M (2015). Outdoor biting by *Anopheles* mosquitoes on Bioko Island does not currently impact on malaria control. Malar J..

[29] WHO (2018). Test procedures for insecticide resistance monitoring in malaria vector mosquitoes.

[30] Griffin JT, Hollingsworth TD, Okell LC, Churcher TS, White M, Hinsley W (2010). Reducing *Plasmodium falciparum* Malaria Transmission in Africa: a Model-Based Evaluation of Intervention Strategies. PLoS Med..

[31] Bürkner P-C (2017). brms: an R Package for Bayesian Multilevel Models Using Stan. J Stat Softw.

[32] R Core Team. R: A language and environment for statistical computing, 2019.

[33] Jindra M, Noret J (2011). Funerals in Africa: Explorations of a Social Phenomenon.

[34] Payne C, Badolo A, Sagnon B, Cox S, Pearson S, Sanon A (2020). Effects of defoliation by the edible caterpillar “chitoumou” (*Cirina butyrospermi*) on harvests of shea (*Vitellaria paradoxa*) and growth of maize (*Zea mays*). Agrofor Syst.

[35] Milali MP, Sikulu-Lord MT, Govella NJ (2017). Bites before and after bedtime can carry a high risk of human malaria infection. Malar J..

[36] Smith DL, McKenzie FE, Snow RW, Hay SI (2007). Revisiting the basic reproductive number for malaria and its implications for malaria control. PLoS Biol.

[37] Midega JT, Smith DL, Olotu A, Mwangangi JM, Nzovu JG, Wambua J (2012). Wind direction and proximity to larval sites determines malaria risk in Kilifi District in Kenya. Nat Commun..

[38] Guelbéogo WM, Goncalves PB, Grignard L, Bradley J, Serme SS, Hellewell J (2018). Variation in natural exposure to *Anopheles* mosquitoes and its effects on malaria transmission. Elife..

[39] Penny MA, Verity R, Bever CA, Sauboin C, Galactionova K, Flasche S (2016). Public health impact and cost-effectiveness of the RTS, S/AS01 malaria vaccine: a systematic comparison of predictions from four mathematical models. Lancet.

[40] Pulford J, Hetzel MW, Bryant M, Siba PM, Mueller I (2011). Reported reasons for not using a mosquito net when one is available: a review of the published literature. Malar J..

[41] Monroe A, Asamoah O, Lam Y, Koenker H, Psychas P, Lynch M (2015). Outdoor-sleeping and other night-time activities in northern Ghana: implications for residual transmission and malaria prevention. Malar J..

[42] Mwesigwa J, Achan J, Luca G, Tanna D, Affara M, Jawara M (2017). Residual malaria transmission dynamics varies across The Gambia despite high coverage of control interventions. PLoS ONE.

[43] Overgaard HJ, Reddy VP, Abaga S, Matias A, Reddy MR, Kulkarni V (2012). Malaria transmission after five years of vector control on Bioko Island, Equatorial Guinea. Parasit Vectors..

[44] Tanga MC, Ngundu WI, Tchouassi PD (2011). Daily survival and human blood index of major malaria vectors associated with oil palm cultivation in Cameroon and their role in malaria transmission. Trop Med Int Health..

[45] Drakeley CJ, Akim NI, Sauerwein RW, Greenwood BM, Targett GA (2000). Estimates of the infectious reservoir of *Plasmodium falciparum* malaria in The Gambia and in Tanzania. Trans R Soc Trop Med Hyg.

[46] Onah MN, Horton S (2018). Male-female differences in households’ resource allocation and decision to seek healthcare in south-eastern Nigeria: results from a mixed methods study. Soc Sci Med.

[47] Dunn CE, Le Mare A, Makungu C (2011). Malaria risk behaviours, socio-cultural practices and rural livelihoods in southern Tanzania: implications for bednet usage. Soc Sci Med.

[48] Moshi IR, Manderson L, Ngowo HS, Mlacha YP, Okumu FO, Mnyone LL (2018). Outdoor malaria transmission risks and social life: a qualitative study in South-Eastern Tanzania. Malar J..

[49] Frey C, Traoré C, De Allegri M, Kouyaté B, Müller O (2006). Compliance of young children with ITN protection in rural Burkina Faso. Malar J..

[50] Choi BCK, Pak AWP (2005). A catalog of biases in questionnaires. Prev Chronic Dis..

[51] Harvey SA, Lam Y, Martin NA, Olórtegui MP (2017). Multiple entries and exits and other complex human patterns of insecticide-treated net use: a possible contributor to residual malaria transmission?. Malar J..

[52] Gryseels C, Durnez L, Gerrets R, Uk S, Suon S, Set S (2015). Re-imagining malaria: heterogeneity of human and mosquito behaviour in relation to residual malaria transmission in Cambodia. Malar J..

[53] Lynch CA, Bruce J, Bhasin A, Roper C, Cox J, Abeku TA (2015). Association between recent internal travel and malaria in Ugandan highland and highland fringe areas. Trop Med Int Health..

[54] Toé KH, Jones CM, N’Fale S, Ismail HM, Dabiré RK, Ranson H (2014). Increased pyrethroid resistance in malaria vectors and decreased bed net effectiveness. Burkina Faso. Emerg Infect Dis..

[55] Hemingway J, Ranson H, Magill A, Kolaczinski J, Fornadel C, Gimnig J (2016). Averting a malaria disaster: will insecticide resistance derail malaria control?. Lancet.

[56] Norris E, Coats J (2017). Current and future repellent technologies: the potential of spatial repellents and their place in mosquito-borne disease control. Int J Environ Res Public Health..

[57] Foy BD, Kobylinski KC, da Silva IM, Rasgon JL, Sylla M (2011). Endectocides for malaria control. Trends Parasitol..

[58] Beier JC, Müller GC, Gu W, Arheart KL, Schlein Y (2012). Attractive toxic sugar bait (ATSB) methods decimate populations of *Anopheles* malaria vectors in arid environments regardless of the local availability of favoured sugar-source blossoms. Malar J..

[59] Dambach P, Schleicher M, Korir P, Ouedraogo S, Dambach J, Sié A (2018). Nightly biting cycles of *Anopheles* species in rural northwestern Burkina Faso. J Med Entomol.

[60] Tusting LS, Thwing J, Sinclair D, Fillinger U, Gimnig J, Bonner KE (2013). Mosquito larval source management for controlling malaria. Cochrane Database Syst Rev.

[61] Burt A, Coulibaly M, Crisanti A, Diabate A, Kayondo JK (2018). Gene drive to reduce malaria transmission in sub-Saharan Africa. J Responsible Innov..

[62] Mutero CM, Mbogo C, Mwangangi J, Imbahale S, Kibe L, Orindi B (2015). An assessment of participatory integrated vector management for malaria control in Kenya. Environ Health Perspect.

[63] Paaijmans KP, Read AF, Thomas MB (2009). Understanding the link between malaria risk and climate. Proc Natl Acad Sci USA.

[64] Obembe A, Popoola KOK, Oduola AO, Awolola ST (2018). Mind the weather: a report on inter-annual variations in entomological data within a rural community under insecticide-treated wall lining installation in Kwara State, Nigeria. Parasit Vectors..

[65] Andronescu LR, Buchwald AG, Coalson JE, Cohee L, Bauleni A, Walldorf JA (2019). Net age, but not integrity, may be associated with decreased protection against *Plasmodium falciparum* infection in southern Malawi. Malar J..

