# Supplementary material for: Quantifying individual variability in exposure risk to mosquito bites in the Cascades region, Burkina Faso
Source: Malar J. 2021 Jan 18;20:44. doi: 10.1186/s12936-020-03538-5 (PMC7814650; doi:10.1186/s12936-020-03538-5)
Supplement: Supplementary file 1 — Additional file 1. S1.1 Interview questions asked of participants. S1.2 Guidelines of topics addressed in informal focus group discussions. S1.3 Template followed for structured observations. S1.4 Summary of methods used to estimate biting risk per person per night. [file 12936_2020_3538_MOESM1_ESM.docx]

# Quantifying the individual variability in people’s exposure to mosquito bites in Burkina Faso

Federica Guglielmo, Antoine Sanou, Thomas Churcher, Heather Ferguson, Hilary Ranson, Ellie Sherrard-Smith

**Supplementary Information 1**

**S1.1 Interview questions guideline**

**Experience of malaria**

Can you tell me what malaria is?

Have you suffered from malaria recently^1^?

Have you had to take care of someone who suffered from malaria recently^[[1]](#footnote-1)^?

How did you/they get malaria?

Why do you think they were exposed to it?

Where did you seek treatment for it?

How long after the first signs of malaria did you seek treatment?

Did the treatment work? Did you have to look for alternative treatment? (Where/How/Why)

**Malaria prevention**

What do you think causes malaria?

Do you think there is a way to prevent malaria? If so, how?

Have you heard of methods of prevention? Have you tried any?

Have you taken part in any sensitisation campaign about malaria?

(If so) What do you remember of it?

Were you the only one from your household to attend?/Why you?

(If not) Do you remember people in the community talking about it?

**Bednets usage and acquisition; Sleeping patterns**

Do you own one or more bednet? (How many? What type?)

Does everyone in your household sleeps under a bednet?

How did your family get them? (E.g. bought, received through distribution campaign)

Can you recall the steps you undertook to obtain a bednet?

Do you sleep underneath it? (Why?)

(If not) Do you use it otherwise?

Do you find it comfortable/convenient? (How so?)

Do you often sleep outside your house (i.e. in the court)?

When this occurs, do you carry a bednet with you?

**S1.2 Focus Group Topic Guide**

1. What is malaria?

Probes: ask the participants to tell, in terms of symptoms, how malaria is different from other illnesses with which it shares the same name (*sumaya*, in Jula); explore any similarities between these illnesses, as well as according to what criteria these are identified and distinguished from one another.

2. Who is more likely to get malaria and why?

Probes: relationship between symptoms and person who falls ill; explore the possibility of changing diagnostics with reference to social status and gender.

3. How do people treat malaria?

Probes: ask what types of treatment are available for malaria (in health facilities as well as elsewhere), and how they differ in cost and accessibility. Aim at bringing to light any relationship that might be identified between symptoms and treatment, and how treatment (in its different forms) can be accessed inside and outside the village.

4. Can malaria be prevented? (how?)

Probes: ask the participants to think about what they know about malaria prevention and how they learnt about it; what is the role of prevention and bednets distribution campaigns; whether they can identify any changes in behaviour (i.e. bednet use) linked to such campaigns.

5. Any Questions

## **S1.3 Structured Observations template**

Conducted every 30 minutes between 18:00 and 06:00 for one week per season (dry/rainy), the structured observations aim at collecting human activity data in a way that is quantifiable and comparable.

Template

Beginning of observation [hh :mm]; End of observation [hh :mm] ; Rain [hh :mm]

| Date Day [Mon, Tue etc] | | | | | | Observation [hh :mm] | | | | | | | | | | | |
| --- | --- | --- | --- | --- | --- | --- | --- | --- | --- | --- | --- | --- | --- | --- | --- | --- | --- |
| ID | Sex | Age | Compound | Household | Guest/  student | 18:00-18:30 | 18:30-19:00 | 19:00-19:30 | 19:30-20:00 | … | … | … | 03:30-04:00 | 04:00-04:30 | 04:30-05:00 | 05:00-05:30 | 05:30-06:00 |
|  |  |  |  |  |  |  |  |  |  |  |  |  |  |  |  |  |  |

| ID | Participant ID. |
| --- | --- |
| Sex | M= male; F= female. |
| Age | In three cyphers. Ex: “23 years old” will become “023”. |
| Compound | Physical residence where the person lives. |
| Household | Nuclear family to which the person belongs. |
| Guest/Student | If there temporarily, note how.  G= guest; S= student; O= other. |
| 18:00-18:30 | Note whether the person is outdoors or indoors every 30 minutes.  1= outdoors; 0= indoors |

Additional notes:

**S1.4 Summary of methods used to estimate biting risk per person per night**

**1.4.1 Estimating outdoor biting risk**

Using the predictable patterns of seasonal densities (Figure S1a) and timing of biting activity (human landing catch HLC data) from across Cascades region ([19], main manuscript) that broadly reflected patterns observed in Niakore (Figure S1b), we estimated the proportion of bites received indoors or outdoors, and the probable number of *An. gambiae s.l.* bites that could be received by a person outdoors in the Cascades region for the weeknights of the year when human activity was monitored in Niakore or Toma (Figure S1b). We assumed that entomological data from one year would still give a reasonable representation of the mosquito activity in the following year, when human behavioural data were collected. We did not investigate whether timing of biting activity patterns (Figure S1b) was altered seasonally.

**Table S1:** The proportion of mosquito bites received indoors (1 – *Φ_O_*, Equation 2 main manuscript) as estimated by the overlapping activity of humans and mosquitoes in Niakore and Toma. These estimates are determined from village-specific data on human activity moving indoors or outdoors throughout a 12-hour period overnight and hourly mosquito blood-feeding behaviour data collected in Niakore in 2016 – 2017. The mean, median and range in the proportion of mosquito bites received indoors for different cohorts of the community are noted.

| Cohort | Niakore | | | Toma | | |
| --- | --- | --- | --- | --- | --- | --- |
|  | Mean (N data) | Median | Range | Mean (N data) | Median | Range |
| Males | 0.79 (14) | 0.84 | (0.54 – 0.90) | 0.70 (19) | 0.72 | (0.51 – 0.83) |
| Females | 0.82 (14) | 0.87 | (0.68 – 0.91) | 0.86 (19) | 0.86 | (0.78 – 0.92) |
| Under 10 years | 0.85 (14) | 0.90 | (0.68 – 0.93) | 0.92 (19) | 0.90 | (0.82 – 0.97) |
| 11 to 20 years | 0.78 (14) | 0.86 | (0.25 – 0.90) | 0.81 (19) | 0.81 | (0.64 – 0.91) |
| 21 to 50 years | 0.81 (14) | 0.82 | (0.72 – 0.90) | 0.75 (19) | 0.76 | (0.58 – 0.89) |
| Over 50 years | NA | NA | NA | 0.80 (19) | 0.81 | (0.68 – 0.89) |
| Oct / Nov 2017 | 0.87 (42) | 0.87 | (0.82 – 0.93) | 0.82 (35) | 0.83 | (0.65 – 0.91) |
| Apr / May 2018 | 0.74 (30) | 0.95 | (0.25 – 0.90) | 0.76 (35) | 0.76 | (0.51 – 0.94) |
| July 2018 | NA | NA | NA | 0.83 (42) | 0.82 | (0.63 – 0.97) |
|  |  |  |  |  |  |  |
|  | Overall | | |  |  |  |
| Males | 0.74 (33) | 0.74 | (0.51 – 0.90) |  |  |  |
| Females | 0.84 (33) | 0.86 | (0.68 – 0.92) |  |  |  |
| Under 10 years | 0.89 (33) | 0.90 | (0.68 – 0.97) |  |  |  |
| 11 to 20 years | 0.79 (33) | 0.81 | (0.25 – 0.91) |  |  |  |
| 21 to 50 years | 0.78 (33) | 0.79 | (0.58 – 0.90) |  |  |  |


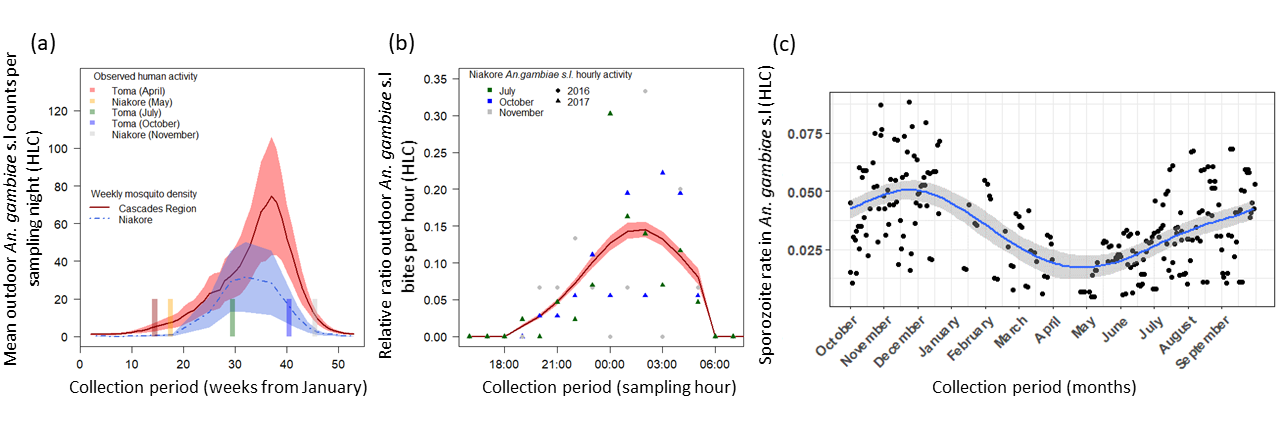


**Supplementary Figure S1:** There is broad agreement in *An. gambiae* s.l. seasonal (a) and hourly biting activity (b) trends in Cascades and Niakore village. Data show the predicted mean and 95% confidence intervals from a generalised additive model (GAM) model where village was considered as an explanatory variable. Model predicted weekly mean mosquito counts are shown for Cascades region (red) and Niakore village (blue). Human landing catch data were collected between 1^st^ October 2016 to 29^th^ December 2019. Here, seasonality was modelled by fitting a non-linear smoothing function on week in the models defined as a scale running from 1 (1^st^ week in January) to 52 (last week in December) with each week of sampling being assigned a value based on the week of collection (collections made on the same week but in different years got the same value). The corresponding week when human activity was recorded in Niakore, May (April) and November (grey), and in Toma, April (orange), July (green), and October (blue) are highlighted. (B) The mean (solid line) and 95% confidence intervals (shaded red polygon) hourly outdoor mosquito biting density across Cascades region as estimated from HLC data (see [19], main manuscript). Niakore specific outdoor mosquito density from HLC data (points) for July 2017, October 2016 and 2017, and November 2016 were highly variable but generally reflected the trend of the Cascades region. For each week of the year when human activity was observed, the mosquito outdoor relative ratio, i.e the number of bites at the specified hour relative to all bites that night (B). *An. gambiae* s.l seeking blood meals were calibrated for the predicted seasonal counts from (A) and (B). For each person, for each hour of human activity observations, the time spent outdoors was multiplied by the estimated number of mosquitoes biting. For each night, these hourly estimates of mosquito bites received were summed to estimate the per night per person exposure to mosquito bites


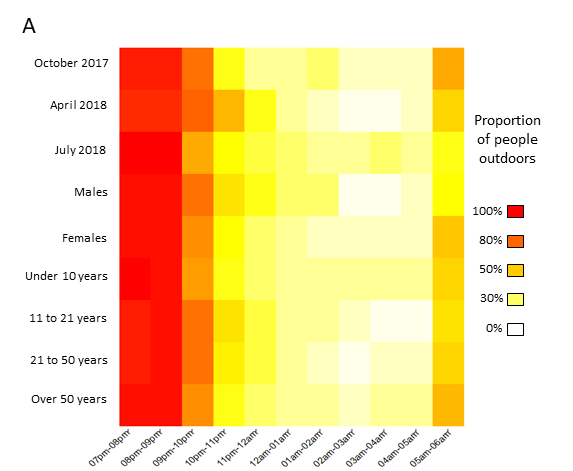


**Supplementary Figure S2.** The proportion of people who are either outdoors (red) or indoors (white) during the night-time hours as observed using passive observations in Toma. Cohorts are distinguished by month (sampling nights in Toma: 8^th^ to 14^th^ October 2017, 6^th^, 8^th^ to 9^th^, 11^th^ to 12^th^ April, or 24^th^ to 30^th^ July 2018), sex (males or females) and age (under 10-years, 11 – 20-years, 21 – 50-years, or over 50-years old).

**
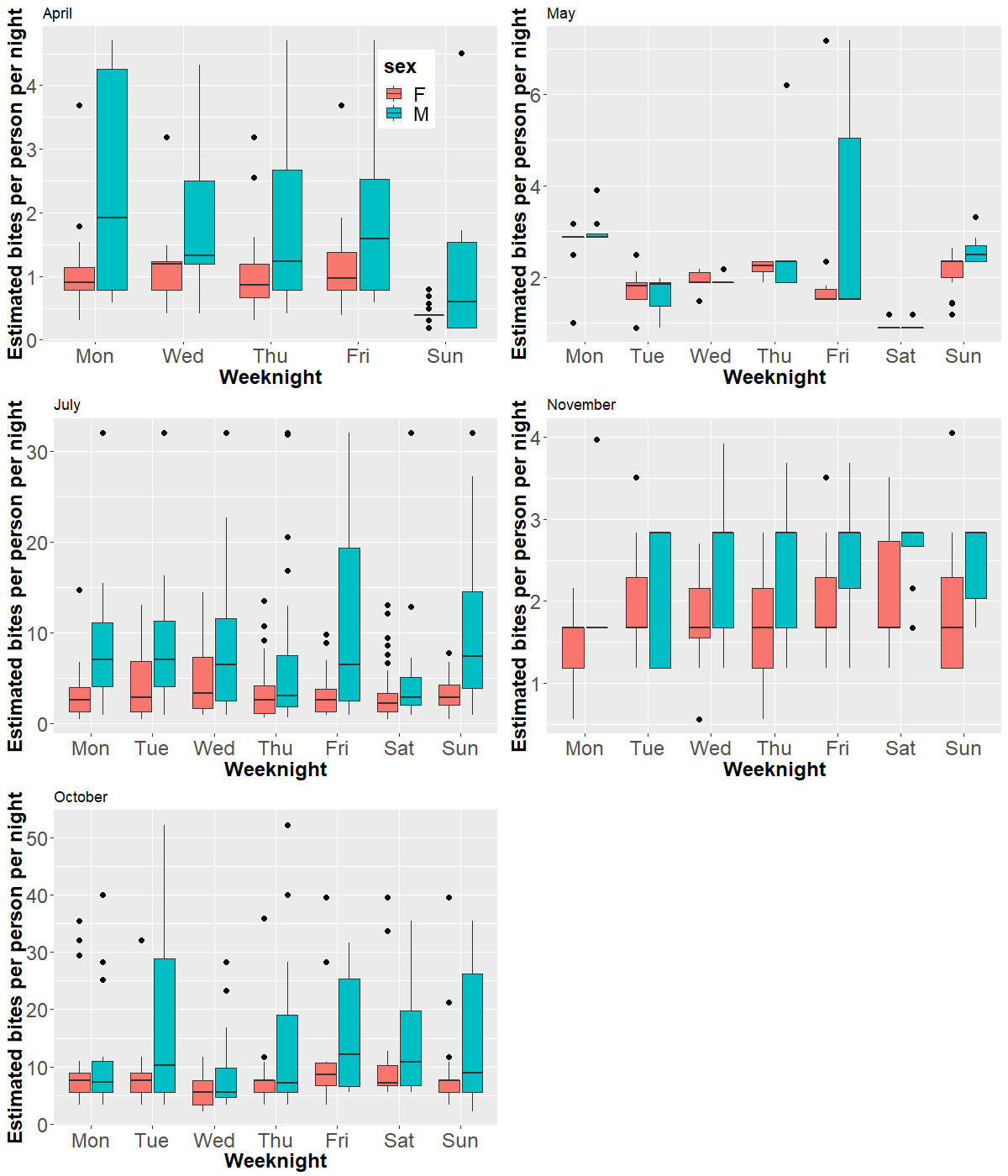
**

**Supplementary Figure S3:** Differences in predicted bites received per person per night of the week that are driven by human activity, numbers show the sample size for each weeknight. Column 1 shows the 3 observation weeks in Toma, column 2 shows the 2 observation weeks for Niakore. Box plots show the median (solid, central line), the lower and upper hinges correspond to the first and third quartiles (the 25th and 75th percentiles), the whiskers extend to +/- 1.5 x the interquartile range for the predicted number of bites per person per night of the week for each month; April, May, July, October and November.

**Table S2:** Univariate analysis of the difference in the predicted bites per person per night of the week. General linear models on log-transformed counts of the predicted number of bites received per person per night of the week were conducted on data for each month independently. Sex was included as explanatory variables, but due to low sample sizes the data were not differentiated further. The villages were sampled in different months.

| Village: Toma | | |  |  |  |  |
| --- | --- | --- | --- | --- | --- | --- |
|  | April:  Odds Ratio | P-value | July:  Odds Ratio | P-value | October:  Odds Ratio | P-value |
|  | Adj-R^2^ = 32.4% |  | Adj-R^2^ = 13.1% |  | Adj-R^2^ = 9.8% |  |
| Monday | Reference |  | Reference |  | Reference |  |
| Tuesday | No data | - | 1.14 (0.86 – 1.52) | 0.360 | 1.09 (0.86 – 1.37) | 0.495 |
| Wednesday | 1.05 (0.83 – 1.32) | 0.680 | 1.23 (0.92 – 1.64) | 0.160 | 0.76 (0.60 – 0.96) | **0.023** |
| Thursday | 0.97 (0.77 – 1.22) | 0.773 | 0.84 (0.63 – 1.12) | 0.248 | 0.95 (0.75 – 1.20) | 0.672 |
| Friday | 1.02 (0.81 – 1.29) | 0.850 | 1.06 (0.79 – 1.41) | 0.711 | 1.14 (0.91 – 1.44) | 0.256 |
| Saturday | No data | - | 0.80 (0.60 – 1.07) | 0.132 | 1.16 (0.92 – 1.46) | 0.210 |
| Sunday | 1.42 (0.34 – 0.54) | **<0.0001** | 1.15 (0.86 – 1.53) | 0.345 | 1.01 (0.80 – 1.27) | 0.950 |
| Sex: Females | Reference |  | Reference |  | Reference |  |
| Sex: Males | 1.59 (1.36 – 1.86) | **<0.0001** | 1.93 (1.64 – 2.25) | **<0.0001** | 1.40 (1.23 – 1.60) | **<0.0001** |

| Village: Niakore | | |  |  |
| --- | --- | --- | --- | --- |
|  | May:  Odds Ratio | P-value | November:  Odds Ratio | P-value |
|  | Adj-R^2^ = 54.3% |  | Adj-R^2^ = % |  |
| Monday | Reference |  | Reference |  |
| Tuesday | 0.58 (0.49 – 0.69) | **<0.0001** | 1.19 (0.97 – 1.46) | 0.093 |
| Wednesday | 0.69 (0.58 – 0.83) | **<0.0001** | 1.17 (0.96 – 1.44) | 0.125 |
| Thursday | 0.81 (0.68 – 0.97) | **0.026** | 1.14 (0.93 – 1.39) | 0.222 |
| Friday | 0.78 (0.65 – 0.93) | **0.007** | 1.30 (1.06 – 1.60) | **0.012** |
| Saturday | 0.33 (0.27 – 0.39) | **<0.0001** | 1.34 (1.09 – 1.65) | **0.005** |
| Sunday | 0.80 (0.67 – 0.96) | **0.017** | 1.24 (1.01 – 1.52) | **0.043** |
| Sex: Females | Reference |  | Reference |  |
| Sex: Males | 1.09 (0.99 – 1.21) | 0.088 | 1.31 (1.17 – 1.46) | **<0.0001** |

1. Adjusted to locally meaningful timeframes. [↑](#footnote-ref-1)
